# Enhancing Reflexivity in Research and Practice in Healthcare Through Oral-Based Autoethnography

**DOI:** 10.1177/10497323241306077

**Published:** 2025-01-28

**Authors:** Christopher Mathieu, Kristine Hagelsteen

**Affiliations:** 1 Department of Sociology, Faculty of Social Sciences, and the Birgit Rausing Centre for Medical Humanities, Faculty of Medicine, 5193 Lund University, Lund, Sweden; 2 Skåne University Hospital & Department of Clinical Sciences, Faculty of Medicine, 5193 Lund University, Lund, Sweden

**Keywords:** autoethnography, reflexivity, healthcare, medical practitioners, academic-practitioner collaboration, orality, autoethnorality, trans-disciplinarity

## Abstract

Autoethnography is an increasingly used method to promote individual and group reflexivity in research, not the least in healthcare. However, autoethnography’s uptake among practitioners is impeded by the fact that it has not been adequately adapted to practitioner settings from its academic origins. This article analyzes the experience of a research team comprised of practitioners/surgeons and social scientists using primarily oral-based autoethnographic practices to promote reflexive collaboration in a longitudinal research and innovation project on selection and training of surgical residents. Based on our case of innovative adaptation and application of autoethnography, which we term autoethnorality, several modifications in autoethnographic practice are suggested to make it more amenable to practitioner settings. These include adopting the collaborative and analytic forms of autoethnography and developing oral-based modalities for autoethnographic practice. The case also shows how these strategic choices along with successive adoption of autoethnographic practices can facilitate the resolution of tensions deriving from the differing timeframes, skillsets, and interests of practitioners on the one hand and academic researchers on the other, as well as paradigmatic differences in theory of science between the medical and social sciences. A table summarizing the advantages and disadvantages of different strategic choices and adaptations regarding autoethnography along with actionable recommendations is presented.

## Introduction

Reflexivity has long been understood as essential to both successful practice and research (Cunliffe, 2004, 2016; Emerson et al., 1987; Schön, 1991). Reflexivity is a means to several key aspects of practice and research by recursively turning thought back on action (Schön, 1991). Reflexivity increases mutual understanding among group members by opening dialogues based on self-understanding (Yang et al., 2020) and facilitates understanding what lies behind objectives and procedures in collaborative undertakings (van Ginkel et al., 2009) and how one’s own knowledge and actions impact both group dynamics and research and action outcomes (Acosta et al., 2015; Miyahara & Fukao, 2022).

Though reflexivity, defined by Cunliffe as “questioning what we, and others, might be taking for granted—what is being said and not said—and examining the impact this has or might have” (Cunliffe, 2016, p. 741), is a cognitive process, it is inextricably linked to practice and action. Silva (2021, p. 1378) writes, “Being reflexive means becoming able to critically reflect on actions and interactions and, based on that, to act in the world.”

Autoethnography is an increasingly used academic method to promote individual and group reflexivity. Autoethnography is a means of gaining insight into one’s embedded relationship with one’s environment through systematic reflecting on one’s own contextual experience through writing (Chang, 2008; Denshire, 2014; Lapadat, 2017). Autoethnography turns *context* from an external phenomenon to be neutrally observed and described into situated probing through the autoethnographer’s experience of it.

Autoethnography’s uptake in healthcare communities has been slower but increasing (Farrell et al., 2015; Netto, 2022; Nowakowski & Sumerau, 2019). Autoethnography is still strongly rooted in the academic world where interests, timeframes, and skillsets are quite different from the world of practice. The rift between the academic and practitioner worlds is often discussed in terms of “rigor” versus “relevance” (Bartunek, 2007; Hodgkinson & Rousseau, 2009; Kieser & Leiner, 2009). Generally, academic horizons are longer, guided by notions of documentation-based rigor, attention to detail, and precision while striving for generalizability. For practitioners, especially in healthcare, relevance and applicability are essential in responding to immediate and practical problem-solving exigencies regarding cases at hand. Successful, immediate intervention is prioritized. When decisions need to be taken quickly, consultation is often *oral* among informed parties (Styhre et al., 2006). Oral communication is superior when urgent work is based on interaction entailing experienced practitioner recall, observation of changing conditions, high case turnover, and cross-case analysis. Though medical occupational activities entail writing and reading texts, much work is via oral interaction. This oral interaction, that is rarely codified, is instrumental for carrying out work tasks, but it also changes perspectives and builds on and develops organizational and processual knowledge undergirding action. As medical practice is already overburdened by documentation, and the majority of oral communication and information is rarely subject to systematic and collective reflexivity, it is pertinent to develop techniques that facilitate reflexive attention to the oral processes that impact how we think and act in healthcare research and practice.

This article argues that an adapted version of collaborative autoethnography utilizing the oral resources and practices established within the healthcare practitioner community which we call “autoethn*orality*” can both bring healthcare practitioners and researchers together when they collaborate, as well as making autoethnoral/graphic processes more applicable for autonomous reflexive use by healthcare professionals.

Empirically, this article explores the opportunities and challenges of developing an “orally-based” form of autoethnography. This is done by self-reflective analysis of the orally-based reflective processes in a case study of an ongoing academic–practitioner collaborative research project consisting of surgeons and social scientists. The project itself is about selection of surgical residents, but the focus in this article is on the processes that have facilitated collaboration and development within the project. We demonstrate how autoethnorality is used to bridge multiple challenges in academic–practitioner collaboration, how autoethnoral/graphic processes go beyond group process dynamics and can generate novel insights about the project context, how oral techniques and procedures are used to perform reflexivity, and the effects these have on individual and collective levels. We also believe our work aligns well with the 2018 Ottawa Consensus Statement on Selection in the Medical Profession, particularly in advocating for more complex, multi-site, and multi-method approaches.

In the next section, we examine recent developments and tensions in autoethnography to explain the virtues of “collaborative analytical autoethnography” we adopt. Then, we discuss methods and data, including orality versus textuality. A brief overview of the collaborative project underlying this article is given before offering empirical examples of how autoethnorality has operated in the collaborative project. The Discussion section focuses on our modified practice of autoethnography and recommendations for adapting autoethnography and autoethnorality to fit healthcare practitioner settings. A table summarizing adaptations and recommendations is presented, and throughout the article, we make references to which rows in this table the current discussion pertains to (e.g., Table 1, row 3). The table synthesizes theoretical points with our empirical analyses. We conclude by highlighting the benefits, challenges, and further potential of autoethnorality/graphy in healthcare practitioner communities and facilitating academic–practitioner collaboration.

**Table 1.** Summary of Autoethnographic (AE) Modifications and Recommendations.

Conventional AE	Our AE Process	Advantages of Our Process	Disadvantages of Our Process	Actionable Recommendations
1. AE as method is known and adopted from outset as part of initial research design.	AE successively acquired over the course of the project, not part of initial design.	As a successive, organic process, AE is not rejected for paradigmatic or practical reasons; it allows for development and use of a familiar modality (orality).	Lack of written documentation—which can impact both reflective analysis and scholarship and publishing.	Depending on the initial degree of acceptance of AE either:
				(a) Introduce AE from the outset, if readily acceptable to the group.
				Then agree on systematic application and generate more text data via less intrusive forms, for example, smartphone voice-recordings or short texts/notes or writing briefs from conversations and meetings.
				Or
				(b) Successively incorporate AE if there is skepticism toward it in the group. To acquire acceptance, display when it is operation and point out the ground gains it achieves. Add more oral or textual techniques over time.
2. Reticence about representing (portraying) and directly impacting others (evocative AE).	Direct engagement with others. The objective of our project is to impact others—improve selection and training of surgical residents; testing and interviewing residents and supervisors entailing powerful scientific portrayals of others and their environments are part of the project methodology and aim.	Engagement with and testing of residents is given by the practical project goal. AE has led to more holistic view of suitability and means of assessment. Interviews are appreciated as a “reflective space,” and testing and training appreciated for technical development by residents. Oral modality entails less durable representations.	Risk of being accused of running afoul of the “problem of representation” and objectification of others. Risk of exercising power over others. Especially collective AE is a means of becoming aware of bias and power in proximate context.	Use AE to become aware of influence on relevant decision makers, goal-setting, and research subjects.
				Keep AE reflection going in dialogue with others, especially those with less power, regarding forms of interaction with them. Report on it in writing and maintain the dialogical imperative associated with practice.
3. Written/textuality.	Orality/verbal.	Orality is fluid, no text or position becomes authoritative; orality is responsive to context and dynamic development; participatory and syncretic. Orality builds on the predominant modality and skillsets in practitioner community.	Continuous narrative lacks documentation and recorded traces—only significant effects are seen, not processual dynamics.	Acknowledge and use oral conversations as information and perspective-carrying resources and investigate how they transmit information and normative perspectives and biases. Keep conversations alive, regular, and link them together. Discuss in conversations *how* things change.
				Complement orality with unobtrusive and convenient documentation—sound recordings, notetaking.
				Regarding the “representation issue,” orality has the advantage of not committing representations of individual and groups to reified text. Representations are ephemeral and readily revisable, not authoritative. Be cautious in writing “authoritative” descriptions of other persons and groups.
				Oral and written notes of discussions can be taken and circulated within the group. Documents produced within the group: meeting minutes, reports, articles, protocols, applications, presentations, etc., can be collected in group “knowledge bank” and made available.
4. “From below”	“From a privileged position.”	An opportunity to improve healthcare practices, for example, selection and training; opportunity to make discoveries only possible from privileged position.	Research group members occupy similar privileged positions: professional qualifications, secure employment, decision-making roles = little status diversity.	Use AE reflection to identify bias, power, and privilege within the group and risks of harm as well as ability to act for improvement.
				Collaborate across status-groups.
				Use privileged position to share knowledge and findings with decision makers and those situated in a less privileged positions through popular science, for example, podcasts, social media, and patient associations’ communication channels.
5. Individual	Collaborative	Synergies of moving from individual to collective levels; views from different disciplines and positions; less risk of becoming self-absorbed/narcissistic; better chance of detecting biases; better coverage of proximate environments.	More difficult to coordinate logistically, cognitively, and normatively; demands time and dedication; risk for compromise rather than unequivocal personal expression.	Advantages of collaboration far outweigh disadvantages; essential in scholar–practitioner undertakings.
6. General institutional contexts.	Collective “ownership” of context, being self-initiated and self-governing.	A strong sense of total responsibility for the context we created and those who participate in it; and for the results and outputs created.	Highly unique context; total ownership of context may lead to defensive dispositions.	Show awareness of and investigate how feelings of responsibility may vary with degrees of “ownership” or commitment.
7. Single discipline even if collaborative.	Different disciplines; academics–practitioner.	Multiple perspectives and disciplinary perspectives become dynamic and change by being challenged; opportunity for scholars to participate in practical improvement, opportunity for practitioners to work with scholar-injected theories, concepts and methods; scholars as scribes in AE activities.	Challenges of overcoming disciplinary and theories of science differences; bridging the scholar–practitioner gap.	Use AE activities—oral and written to bridge gaps and facilitate mutual learning by also focusing on how challenge and learning is experienced.
				New knowledge is created in the cross-section between disciplines and paradigms.
8. Evocative, interpretivist/constructivist paradigm.	Analytic, realist paradigm.	Realism lies closer to scientific and practitioner perspectives and commitments in our research group; analysis of context (through personal experience) is key to interventions/action in context.	Possible impression that one is “doing AE incorrectly” as analytic AE is still a minority and from some quarters condemned branch of AE.	The scientific paradigm should fit the ambition and point of departure of the project and project members (academic and practitioners), but be open for challenge and development.

## Background

### Autoethnography

Autoethnography has grown rapidly in recent decades and different conceptualizations of it exist. There is though consensus regarding central features of autoethnography (O’Byrne, 2007). The first pertains to the “auto” or *self* in the concept. This privileges and validates the researcher’s own personal subjective experience as the central theme and data source in accounts, leading to increased visibility of the first-person author in the text. The second pertains to “ethno,” the ethnos, culture, or group context that the autoethnographer has direct experience of, reflects over, and gains deeper insight into. In some autoethnographic studies, the ethnos is a specific social community (e.g., a ward), in others it is a wider, diffuse categorical group or culture. In distinction to classical ethnography, where the ethnographer is an outsider, autoethnographers are insiders, or in Anderson’s (2006, p. 379) terms, a “complete member in the social world under study.” The final component “graphy” refers to the role of writing and text in two regards. Most autoethnographic practice is carried out through producing and reflecting on one’s own written accounts of personal experience in a specific context. Douglas and Carless (2013a, p. 89) go so far as talking about the “textual self” in autoethnography, directly connecting *auto* and *graphy*. The second regard points to the fact that the output of autoethnographic activity is a written document. Autoethnography “refer[s] to the research process as well as the written report, story or performance that is produced” (Lapadat, 2017, p. 590). Chang (2016) summarizes autoethnography as: “a qualitative research method that uses a researcher’s autobiographical experiences as primary data to analyze and interpret the sociocultural meanings of such experiences. The ultimate goal of autoethnography is to connect ‘the personal’ with ‘the social’” (p. 444).

Despite these basic agreements, major tensions within the method exist, not least over theory of science issues and who should be (re)presented in texts.

#### Analytical Versus Evocative Autoethnography

From its inception, autoethnography has been rooted in interpretivist and constructivist theories of science (Denzin, 1997; Ellis & Bochner, 2006), divorcing itself from classical realist ethnography (Lofland, 1995). The interpretivist tradition rejects the idea that social phenomena can be explained in terms of causal processes, seeking rather to understand social phenomena in terms of the meaning they have for their participants (Table 1, row 8). While much research in the interpretivist tradition seeks to understand and portray the meanings of others, evocative autoethnography retreats even from this on ethical and epistemological grounds—the so-called “problem of representation”—and focuses solely on the meaning and experience of the autoethnographer her/him/itself (Table 1, row 2).

Anderson’s (2006) analytical autoethnography directly challenges both its rooting in interpretivist/constructivist epistemology and focus on the autoethnographer’s subjectivity (Table 1, row 8). Anderson contends that to be analytical, autoethnography should be rooted in a realist paradigm (Lofland, 1995). Furthermore, Anderson (2006, pp. 386–387) argues autoethnography should *analyze* the world “beyond the self,” necessitating representing others in autoethnographies (Table 1, row 2).

The rift between the “two branches” (Bunde-Birouste et al., 2019, p. 510) continues to be debated. Hughes and Pennington’s (2017) practical guide to autoethnography declares analytic autoethnography a central and distinct approach with a different (realist) ontology and legitimacy basis, while Ellis et al. (2011) write analytical autoethnography out of the history of autoethnography, except as an external criticism of autoethnography by adherents of traditional ethnography.

#### Individual and Collaborative Autoethnography

A major advance in autoethnography is its extension from an individual to a group undertaking (Table 1, row 5). The term “collaborative autoethnography (CAE)” is applied when two or more persons collaborate in analyzing and reporting their experiences (Chang et al., 2013; Lapadat, 2017). CAE poses challenges but has several advantages over individual autoethnography. Acknowledging the epistemological position shared across autoethnography that “any account they [reflexive researchers] provide is a partial perspective as seen through their own point of view at a particular time and place” (Lapadat, 2017, p. 591), Miyahara and Fukao (2022) argue “CAE contributes toward minimisation of [positional] shortcomings through dialogues among the researchers, so as to counter-balance the researchers’ subjectivity” (p. 3). Validity, a non-issue for evocative autoethnographers, is explicitly addressed by Lapadat (2017): “Part of the concern about [autoethnography]’s focus on the self is that as a method, autoethnography has no means of auditing the data construction or checking the validity of the self-analysis” (p. 596). Lapadat (2017) finds that collaborative autoethnography affords greater rigor than individual autoethnography, as when “Two or more researchers contribute to data generation … different disciplinary and experiential perspectives … can deepen the analytical and interpretive components” (p. 598) (Table 1, row 7). While collaborative autoethnography can be conducted within both evocative and analytic approaches, jointly involving multiple researchers allows for concerns at the heart of realist social science to be more readily addressed through the collaborative structure. This convergence has led to collaborative *analytic* autoethnography (CAAE) becoming a subgenre itself (Acosta et al., 2015).

Lapadat (2017) also identifies epistemological and pragmatic challenges with collaborative autoethnography: “The success of such collaboration rests on the ability of the coresearchers to negotiate personal dynamics and cultural elements, as well as different goals, timelines, and kinds of knowledge that members of the team bring to the experience” (p. 591). While personal dynamics, cultural and knowledge differences, and goals may initially vary and clash, as we show regarding our own case, orally-based CAAE itself is an effective means of handling such initial divergences (Table 1, row 7).

### Autoethnography in Practitioner Settings

Practitioner autoethnography gives both insight into the experiential dimension of work and knowledge and motivational foundations for bottom-up interventions and change in workplace systems (Bunde-Birouste et al., 2019) (Table 1, row 4). On the first point, Denshire (2014) argues that autoethnography contributes to more balanced accounts of practitioner work, as “An auto-ethnographic representation of practice can function as something of a corrective to depersonalized and disembodied accounts of professional work” (pp. 835–836). Combining both points, Acosta et al. (2015) argue that autoethnography, especially CAAE, is a highly appropriate technique for action research as,

the practitioner-researcher aims at action (change) and understanding (theoretical connections) as practiced by “insiders.” They are members of the professional culture and setting they are investigating. … CAAE supports dialogic communication, which promotes theoretical understanding and theoretically-informed decision-making. … Finally, autoethnography provides a research tool that allows practitioner-researchers to investigate a problem, or topic, related to their practice … systematically. (p. 414)

Moving from the content of occupational practice to group dynamics, Lapadat (2017, p. 599) finds that especially collaborative autoethnography has positive effects on cooperation as it “flattens power dynamics,” as in collaborative autoethnographic undertakings “all are coresearchers” with the validity of personal experience having an equalizing effect.

### Autoethnography in Healthcare

Autoethnography is increasingly used in healthcare research (Farrell et al., 2015; Netto, 2022; Nowakowski & Sumerau, 2019), generally taking two forms. One is deep, personal, and qualitative description of patients’ experience of illness, their treatment, and healthcare systems (O’Connell, 2023; Uotinen, 2011). The second is personal and qualitative description of healthcare professionals’ occupational and emotional experiences about working in healthcare systems or medical education (Farrell et al., 2015; Ibrahim et al., 2023). In some cases, these two genres are combined, where healthcare professionals produce autoethnographic studies of their own illness, often refracted through medical professional understandings of illness and healthcare systems (Liggins et al., 2013). What is less common, and our empirical focus, is using autoethnography to generate reflexion within a research group exploring a core facet of the healthcare system (Kestenbaum et al., 2015; Russ et al., 2013). As our research is on the social phenomenon of personnel selection and training of surgeons, and not diagnostic procedure or disease treatment, it is appropriate to use autoethnography as a tool to increase awareness of group dynamics and the research process, and reflect on the knowledge and power that the project group wields within its proximate and extended context (Table 1, row 2, row 4).

The literature demonstrates how the two primary strands of autoethnography, evocative and analytic, lead to different trajectories (Table 1, row 8). Analytic ethnography with its realist foundations and orientation “beyond the self” is more embraced in collaborative, practitioner settings, while evocative autoethnography remains predominant in the academic sphere where autoethnography originated.

## Methods and Data

### Setting: The Collaborative Academic–Medical Practitioner Project

To understand our autoethnographic process, it is important to understand the nature and setting of our project. The research project is self-initiated, self-governing, and practitioner-driven (initiated and led by surgeons) (Table 1, row 6). Its aim is to improve procedures for selecting and training surgical medical residents in Sweden’s decentralized system (Hagelsteen et al., 2021).

The project follows 50 surgical residents over their 5-year residency program. The residents are recruited from southern Sweden and called to “test-days” four times: (1) when interviewed or hired for residency; (2) after 1 year of residency; (3) in their third year; and (4) after their fifth year when they have completed their residency. During “test-days,” residents are interviewed with standardized bespoke interview guides unique for each year-level. Questions focus on personal motivation, progress, and their work and learning environment. At each test-day, one to four of the surgeon project members are present, carrying out various laparoscopic testing activities, and two sociologists conduct the interviews. Surgeons participated in year 0 and 1 interviews, whereafter the sociologists conducted the interviews on their own to allow the residents to speak more freely about their experiences without surgeon colleagues present. The residents are also tested and trained on two laparoscopic surgical simulators at each test-day. Personality self-assessment and visuospatial tests are done online off-site in the first and final year. The residents’ supervisors are interviewed in the resident’s fifth year. Resident interviews and tests have been conducted since November 2017, with the final cohort enrolled in Spring (2021) (*n* = 50). As the study runs over full residency periods, the project will run through 2027. Approximately 15 test-days are held annually with 1–4 residents per day, meeting approximately 30–35 residents annually. Regular but temporally interspersed contact with the residents means that a feature of each test-day is discussing project developments since the last meeting with them. These discussions take place when we convene in the morning, during breaks, and especially over lunch when all the residents and 3–4 project members at the test-day eat lunch together (see the “campfire history” below).

### Project Members, Funding, and Ethical Clearance

The project team comprises of physicians and social scientists: four clinically active consultant surgeons (all MDs and PhDs, experienced in surgical education and simulation training and assessment); two organizational sociologists (PhDs); and one psychologist (PhD). Two MDs have joined the project as PhD students. Project members are jointly responsible for data generation, analysis, and reporting results.

Initial project funding came from regional government and hospital funds. Current funding is provided by the Swedish Prostate Cancer Society and the Swedish Patient Insurance Agency (Löf—regionernas ömsesidiga försäkringsbolag). The variety of funding sources affirms the project’s independence and patient-safety orientation. Ethical approval was obtained from the Swedish Ethical Review Authority (approval number: EPN 2016/1050, with amendments 2022-04361-02 and 2023-04850-02). All data is de-identified and treated confidentially, available only to research group members and *not shared with employers*. All participants gave informed consent to participate.

### Orality as Context and Method

As orality is the primary modality in our “autoethno” process, oral sources and transactive memory are central as data (Table 1, row 3). As Furniss (2004), Ong (1982), and Silva (2021) show, orality is a comprehensive means of developing and bearing self- and contextual understandings in public narratives. This is not the same as storytelling in organizations. As Gabriel (2000) points out, “not all narratives are stories; in particular, factual or descriptive accounts of events that aspire at objectivity rather than emotional effect must not be treated as stories” (p. 5). We therefore refer to our oral data as histories rather than stories. Orality is more comprehensive than storytelling in terms of genres contained, skills entailed, and amount and quality of information conveyed, referenced, and stored. As Ong (1982) writes, where orality is the central modality, “narrative is particularly important in primary oral cultures because it can bond a great deal of lore in relatively substantial, lengthy forms that are reasonably durable” (p. 138). Our project’s running narrative (“campfire history,” see below) operates in the manner Ong describes above, being developed and recounted collectively over 15 times annually at test-days and project meetings. It incorporates new discoveries alongside or sometimes in contrast with previous thinking and formulations, providing a current account, while also having previous accounts at its disposal. This is especially the case when many project members and participants are engaged in the narrative discussion, as they can bring in novel and historical matters they find significant from their individual perspectives.

Importantly, the running narrative also becomes codified in funding applications, reports, meeting notes, protocols, and academic publications. In this hybrid manner, orality and textuality complement each other. The narrative provides contemporary content for these strategic documents, while these documents provide codified historical snapshots of project processes and thinking that can be used to check and augment the running narrative (Table 1, row 1, row 3).

Socially, orality and textuality function differently. Orality, Furniss (2004) argues, draws people together in discussion and is thereby more open and dynamic, whereas textuality isolates, both socially and cognitively. This is why orality is the central modality in our collaborative research process, as well as in healthcare practice, where considerations often require input from many quarters, patients and relatives, physicians, nurses, physical therapists, etc., and often in an immediate timeframe. Ong (1982) would concur with Furniss, while pointing out a different affordance, in that the written word “asserts itself with finality and authority with an object permanence” making it central in academic pursuits. In line with the reasoning of Ong and Furniss, Lapadat (2017) sees a danger in autoethnography having writing as its central modality: “once an autoethnographic story has been written, the written version has the effect of reifying that particular story. An autoethnographer’s point of view and understanding will change over time, but the text persists, frozen in time, and tends to be read as an authoritative account” (p. 594). As argued above, the virtue of orality is that it keeps pace with relevance, is less burdensome than writing, and matches the verbal communication skillset of healthcare practitioners. Its disadvantage is what may get lost in revision. This summarizes the primary advantages, as well as disadvantage of autoethnorality.

### Data Generation and Analysis

The methodological foundation for this article derives from CAAE as outlined above. This has implications for our data sources, analysis, and presentation, especially as our analysis centers on the ephemeral oral modality of our autoethnographic process. In keeping with autoethnography, data sources for this article are *self-generated*, comprising both written reflective accounts, and significantly our continuous running-account oral history and transactive memory (Ren & Argote, 2011; Yan et al., 2021) of the project process. These data sources are auto-reflectively generated by project members, fusing personal experience with analysis and effect outcomes. In keeping with autoethnography, data presentation, analysis, and effects or outcomes are inextricably linked in personal accounts.

The primary methodological challenge for this article is taking something that is by its oral modality dynamic, dialogical, and ephemeral, and rendering it in academic text, which is static and authoritative (Lapadat, 2017; Ong, 1982). To accomplish this, we use two approaches to data analysis and presentation for “textualizing” oral sources and memory (Silva, 2021). The first is conventional autoethnographic written retrospective narratives (Denshire, 2014; Hughes & Pennington, 2017). A truncated excerpt by one of the surgeons is presented below, as well as an account of how autoethnorality provided the foundations for writing a guidebook for surgeons-in-training. The second is collaborative analytical accounts by the two article authors of specific significant episodes from the almost decade-long project history.

We illustrate how we adopted and adapted autoethnographic techniques in our collaboration to promote reflexive understandings. These assisted us in dealing with our conjunctive challenge: getting a multi-disciplinary research group to: (1) understand each other and (2) understand how the nature of the project, procedures, and methodologies are seen and acted upon differently from the individual, disciplinary, and theories of science perspectives represented in the project group (Table 1, row 7). Applying autoethnorality brokered methodological differences and facilitated gaining insight into the operation of our project and the wider context of resident selection and training.

Below we present four specific moments and outcomes displaying different applications of the autoethnoral/graphic processes. First, a truncated reflective narrative by a surgeon project member describes how autoethnographic dialogue mediated a rapprochement between divergent scientific paradigms. Second, we show how reading a key autoethnographic text (though not labelled as such) both facilitated the abovementioned paradigmatic rapprochement and paved the way for our understanding and embracing of autoethnography as a method for scientifically understanding our own process and context (Table 1, row 1). Third, we analyze how the project’s dynamic ongoing oral history serves as a continuous space for both internal reflexion and external communication (Table 1, row 3). Fourth, a narrative from the project leader (second author) explains the reasons for publishing a handbook for aspiring surgeons and supervisors on the themes arising during the research process and our scientific discussions over the years (Frost & Hagelsteen, 2024) (Table 1, row 2, row 4).

## Process Descriptions, Analyses, and Results

### Our Autoethnographic Journey: A Narrative

The truncated retrospective reflexion below written as an autoethnographic exercise by one of the surgeons mirrors in a condensed manner at the individual level the evolution of the project. Several important topics regarding the intertwining of project and personal development are brought together, especially linking science paradigms to understandings of suitability.

It wasn’t easy for a humble surgeon, who prefers to see and measure the world in numbers and statistically significant p-values, to start reflecting upon the unspoken secrets in the art of being a physician. The project originated in simple studies of learning and execution of laparoscopy,^1^ where the study participants could be evaluated and graded. From this, a discussion arose that some surgical trainees have difficulty learning the technical aspects of surgery. This discussion expanded further to more soft issues, as we (surgeons) all experienced colleagues who are less suitable (but there are also many who are very suitable) for the occupation. The less suitable can be careless, indecisive, risk-takers, moody, have difficulty with social interaction, lack good communication tone, etc. Those who are deemed suitable are willing to work, punctual, conscientious, adequately decisive, communicate well, empathetic and aware of their own limitations. To gain more insight and reflect upon what can be understood as a good/less good surgeon, we conducted interviews with senior surgeons and heads of departments to gain more material for reflection as well as autoethnographic work within the research group. This collaboration has, for the medical professionals made it clear that orthodox medical science needs to be complemented with non-quantifiable qualitative knowledge. Through our autoethnographic discussions it became clear that becoming a complete surgeon in all facets, cannot be quantified, it requires deeper qualitative reflection and analysis.

As stated, autoethnographic activities became the means of attaining greater insight into both the content and process of assessing suitability, the core of our project. The narrative charts successive realization that non-quantifiable qualitative knowledge (from oneself and medical peers) is essential for gaining insight into dimensions beyond technical capability, where the project started. As the project moved away from the comfort zone of orthodox medical science, individual introspection and collective self-reflection enabled by autoethnographic inquiry increases in significance. Although the passage above is a *written* extract from a prompted autoethnographic exercise, it names the primacy of orality and discussion as the primary vehicle in this journey.

### Encountering an Autoethnographic Account Before Encountering *Autoethnography*

While engaging in dialogic autoethnoral reflection from the very beginning of the project, our first exposure to an autoethnographic text (thought this label is not used by the author) occurred over a year into the project (Table 1, row 1). The project leader, a surgeon, suggested reading Kneebone’s 2002 article “Total Internal Reflection: an essay on paradigms,” which resonated with her feelings and experiences within our project. Kneebone describes his initial encounter with social science and humanities perspectives thus:

I am a doctor in my mid-forties, with a background in surgery and general practice. Recently I have begun working towards a thesis on surgical training and have started to explore a new literature – that of education and the social sciences … I found this literature almost impenetrable … My difficulties lay somehow at a deeper level, one I at first found hard to articulate. I had the disquieting sensation of moving into alien territory, where familiar landmarks had disappeared. … I believe that my difficulties were caused by a clash of world views – or rather, a clash between the comforting solidity of orthodox “science” and the fluidity of those disciplines which challenge their own paradigms as a matter of course. … The fact that I have practised as a doctor for more than 20 years without being forced to confront these issues makes a telling point, not only about me but about medical education more generally. It seems to me that our system of medical training places formidable obstacles in the path of those who choose to move outside it. (Kneebone, 2002, p. 514)

For the physicians, the article provided a frame of reference and validation of their experience in encountering qualitative social science (Table 1, row 7). For the social scientists, it provided a rich personal description of a journey from mainstream medical science to the social sciences. For both groups, it provided a common vocabulary and reference point for ongoing discussions about methods and theories of science, understanding our ongoing discussions since the inception of the project, and the prospects for reconciling the medical and social scientific approaches. Beyond its content, Kneebone’s (2002) article was central to our ultimate embrace of autoethnography (Table 1, row 1) as it displayed autoethnography’s practical relevance in guiding our discussions and legitimating introspection and public discussion of what can be construed as strictly personal experiences of “shortcomings.” Shortcomings Kneebone furthermore shows to be rooted in professional educational shortcomings (Table 1, row 7).

### The Dynamic Oral History: An Evolving Campfire History

Our primary reflexive space is our narrative oral-history description of the project (Table 1, row 3). It is used and developed in various forums, most often test-days, but also internal project meetings and external presentations. It variously comprises the origins of the project, usually a developmental tale about how its original objectives and points of departure have been modified updated and why (see the surgeon’s narrative above); how we as researchers and practitioners have developed through the project; how our interpretation of the process and preliminary results have changed over time; what our major achievements are; what our current and past tensions and points of conflicts are; how our perceptions have changed regarding the healthcare environments we hear about and work in; and what we hope to ultimately achieve through the project. This “campfire history” gets told and updated in different variants roughly twice a month—when meeting project participants on test-days, at internal project meetings, public academic and popular science presentations, and presentations to our funders. Most often, it is elaborated in collective settings on test-days, where between one and four residents meet with two to five project members (always at least one surgeon and one sociologist). The narrative is interactive, usually entailing current thinking and approaches contrasted with previous ideas, recent activities, and accomplishments coupled with questions from the residents prompting innovative formulations and discussion among the project members. These conversations can last from 10 minutes to an hour over lunch. Sometimes it contains only minor variations on known themes, and sometimes novel formulations and insights and ideas emerge important enough to be discussed by all present and retained and disseminated to non-present members of the project group orally or by email. These novelties become incorporated into the next iteration of the project oral history.

These oral accounts provide the primary content for and are further developed in writing accounts of project activities and accomplishments in reports to financiers, academic publications (as direct below), and presentations to the medical community at conferences and invited engagements. In other words, this well-laden, well-versed, oral history is the primary resource we draw upon in both formal and informal contact with internal and external stakeholders.

### A Handbook for Aspiring Surgeons and Their Supervisors

Recently, the project leader (surgeon, second author) published a handbook for trainee surgeons and supervisors (Frost & Hagelsteen, 2024). The subjects covered in the book consist of shared knowledge and themes emerging from discussions within the research group, not least the ongoing project history. The themes also arose from conversations with colleagues, residents, and patients, as well as notes from podcasts and relevant performance literature. This handbook shows how oral sources can be “textualized” for wider dissemination and practical guidance. The author’s reasoning for writing the book is recounted:

Over the years since I published my dissertation, which addressed, among other things, the issue of unsuitable surgeons, I observed a growing interest in this topic. I receive numerous requests to share my expertise, with the expectation or hope that I have a “solution to the problem.” Gradually, I accumulated brief notes based on conversations within and outside the project, written in various places and realized that I had enough material for a handbook aimed at future surgeons and their supervisors. Together with a colleague who shared an interest in performance and competence from a broader perspective, we wrote a book. The scientific discussions within our research group, combined with the concrete examples that my colleague and I, both experienced program directors, shared, led us to believe that we have partially addressed the need for a “solution.” In the scientific literature, the topics we cover in the book are referred to as “the hidden curriculum,” knowledge that emerges through the experience of being a surgical resident. I hope the book can function as a companion and alleviate some of the insecurity everyone feels being new at a surgical department, and thereby normalize the thoughts on one’s own suitability and professional development.

The autoethnographic action of data collection, converting conversation to notes using a smartphone’s notetaking application, is an example of how written documentation can be harvested from oral sources. This hybrid form of oral and written notetaking of oral source material was unobtrusively done on a phone and paper (Table 1, row 1, row 3). This process was done continuously from 2018 in different folders containing several short notes named, for example, “wise stuff,” “better,” “thoughts,” and “presentations.” The notes were created using voice-recording after meetings, telephone conversations, test-days, and conversations with participants, as well as written text quotes picked up in the academic literature and from public and media discussions outside the research context.

This is one technique for “textualizing” autoethnorality—first in terms of notes derived from conversations and oral histories, then in turn formalized into a guidebook. Notes were referred to as part of “the hidden curriculum.” Noteworthy is that the “hidden curriculum” is primarily experienced and transmitted orally, and only occasionally codified in writing. The handbook is an example of how certain forms of knowledge exist predominantly in oral or textual modalities but also how the oral can be captured and rendered in text form. Example of notes and how it translated into text in the book are given below.

Note: Work culture and the golden rule.

Book (Frost & Hagelsteen, 2024): Navigating without sharp elbows. Excellence is a team performance. When you are new at the workplace, you quickly notice the culture among your colleagues. Soon enough, you become a part of that culture. Strive to be your best and contribute to creating the kind of culture and atmosphere you would like to be welcomed into. Be generous. You will imitate those around you. Become someone that contributes to team development. You have a common goal.

Note: Attention to detail is a key point when learning and performing a procedure.

Book (Frost & Hagelsteen, 2024): Talk about technique with your colleagues. When you drink coffee, during surgery. Share something you’ve learnt and ask their opinion. Ask the colleague to share something he/she learnt.

Note: Imposter phenomena

Book (Frost & Hagelsteen, 2024): The impostor phenomenon was coined in 1978 to describe the profound sense of self-doubt and fear of being exposed as a fraud experienced by high-achieving individuals, despite objective measures of success. Numerous studies indicate that the impostor phenomenon is prevalent among surgical residents.

As a physician at the beginning of your career, you must rely on your acquired medical knowledge and practiced skills as a foundation. Everyone has been new to the job at some point. Remember that patients do not always expect you to have all the answers immediately. Seeking advice from more experienced colleagues does not imply that you are an impostor; rather, it shows that you recognize your limitations, which is appreciated by both patients and the organization.

The reception of the book upon release at a conference arranged by the Swedish surgical residents’ society confirmed the authors’ perception regarding the demand for these types of conversations, which can be catalyzed by converting the oral-based information exchanged for years into codified form.

## Discussion

In this section, we analyze our process to extract specific lessons about how autoethnography can be modified and used to promote reflexivity in practitioner settings and academic–practitioner collaboration.

### Autoethnography for the Benefit of Group Dynamics

In collaborative undertakings, autoethnography can profoundly improve group dynamics. Our autoethnorality process reduced two sets of differences. The first being between full-time academics and predominantly clinical practitioners and the second being between those trained in medical versus social sciences. Differences were reduced to the personal-experience level, thereby making these negotiable rather than matters of principle. This was facilitated by making two opposing experiences, encountering novelty and affirmation of expertise, the basis for confronting major issues from a position of equality. All project members were simultaneously experts in their respective fields but quickly realized that this expertise was only partial to the collective endeavor; we all had to move into very novel territory (Table 1, row 7). We drew on each other’s expertise, and all had the common personal experience of being curious neophytes in worlds quite different from what we were schooled in (Kneebone, 2002). This goes beyond what Lapadat (2017, p. 599) calls the “flattening of status” in autoethnography by making everyone “co-researchers.” Autoethnography provides a basic platform of equality not just in terms of being both novice and expert but at a more fundamental level by affirming the legitimacy of one’s own experience as a basis for interaction. This bridges initial knowledge gaps and incommensurabilities. From here, it is possible to discuss issues such as theories of science, perceptions of suitability, measurement techniques, and work-worlds through personal experience in an open and equitable manner as reported in the surgeon’s narrative above.

### Adopting and Adapting Autoethnography

How and when autoethnography is adopted impacts how it can be modified and adapted (Table 1, row 1). Autoethnography can be selected a priori as a primary component in research design, guiding the entire process. Or, it can be subsequently adopted after a study is initiated in varying degrees of formality as a complementary support technique for accomplishing other objectives, such as increasing reflexivity. In our case, subsequent adoption allowed for practicing autoethnography even before labelling it as such, adapting it to our conditions without feelings of transgressing autoethnography’s methodological orthodoxy. We employed an organic dialogic autoethnoral process from the outset to deal with challenges of integrating the multi-disciplinary research team and negotiating objectives and procedures arising from being a self-initiated, self-governing project (Table 1, row 6). Experiencing the utility of Kneebone’s text facilitated our dialogue dealing with different scientific paradigms, displayed the usefulness of autoethnographic thinking, and solidified our oral performative approach to autoethnography/orality (Douglas & Carless, 2013b). When autoethnography was subsequently introduced to the group as a formal methodology, we already had enough positive experience with the oral modality of autoethnographic reflection to accept and work with orality as our primary reflexive medium, especially after recognizing our running project narrative as a functional equivalent to reflective writing. Once formally incorporating autoethnography, we also began using explicit written reflections, of which the narratives above are truncated examples.

Successively discovering and implementing autoethnography has benefits and drawbacks (Table 1, row 1). The most important benefit is giving acceptance of autoethnography an opportunity to grow over time. It is quite likely that if suggested at the outset of the project, when the differences between the classical medical scientific paradigm and qualitative social science perspectives were still in their primal unbrokered states within the project group, autoethnography would have been dismissed out of hand by a majority of members of the group as “unscientific”—subjective, unquantifiable, and lacking a control-group. When ultimately recognized and introduced as a formal methodology, it had proven its practical value in mediating the challenges of multi-disciplinary research, promoting positive group dynamics, and developing in the oral modality close to healthcare professions rather than requiring cumbersome writing activities. Our adaptation was expedient, and orally-based autoethnography was recognized as something we had been doing almost from the outset. The successive route gave us the opportunity to experience its value before applying the label and uniquely adapt autoethnography to our conditions, needs, and abilities. We believe that other multi-disciplinary research collaborations like ours can benefit from our experience and adopt similar ways of working and develop their cooperation and understanding of each other’s competence and knowledge. The major drawback associated with the predominant use of orality in our autoethnographic process is a lack of formal codified documentation of the content and process itself, which could also provide an extra dimension of input in our dialogical process and charting our development.

As shown above regarding how the oral history became the basis for various documents, presentations, and the handbook, even orally-based “autoethno” processes can advantageously operate in a hybrid manner. Unobtrusive notetaking using telephones’ voice-recording and written notetaking functions or pen and paper notes allows ideas and direct quotes to be saved verbatim and in context. Presentations and minutes or memos from meetings can support the oral process or become autonomous sources of reflexion. Formal and informal documents are inevitably produced even in largely orally-based contemporary work-settings, and building and making available a digital “knowledge bank” of such personal and collective documents, including presentations, articles, email correspondence, and other group records, can be invaluable, especially when incorporating new group-members (Table 1, row 1, row 3).

### Different Roles Regarding Autoethnography

Despite acting as an integrated research unit, we find that the social scientists and medical practitioners play distinct roles in relation to our autoethnoral/graphic practice. The social scientists have not just formally introduced autoethnography but also theorize the approach in terms of project objectives and processes. This includes explaining its relevance for the project, what benefits it can provide to the research group and project development, and making equivalencies between our practical activities and how autoethnography is described in the literature. As noted above, this was made easier by the research group early on finding Kneebone’s (2002) article useful. The social scientists also provided the confidence to modify and break with key elements of the methodology, in particular promoting orality as our primary modality. The practitioners applied autoethnography not just in the context of the project but also to their experiences as surgeons, members of the medical profession, and products of medical scientific training. Relevance and application were discussed within the group, leading to new directions and procedures. This included modifying interview guides to not just focus on measurable responses at the individual level such as which operations are conducted entirely by the resident to questions probing the work and learning environment; how different types of tests are juxtaposed in terms of correlations and mediating variables; and interpreting results taking wider contextual factors and individual development trajectories into consideration rather than merely attained values.

### Insights From the Autoethnographic Process

Autoethnographic insight also led to a shift in understanding the core concept of suitability and how it can be assessed. The surgeon’s narrative reflection above charts a shift from the project as a collection of quantitative test instruments to where we are now. This development, also embedded in the continuously evolving project “campfire history” is referred to by the project leader in a notebook entry 4 years into the project. She terms it as progressing from an initial conceptualization of the project as “The science of finding future surgeons through triangulation” to “The art of navigating between pitfalls in hiring surgeons.” This marks a shift from questing for the one best selection method to a realization that there may be different positive means, and emphasis should be on creating a system to avoid costly recruiting mistakes.

### Structural Factors Impacting and Supporting Our Autoethnographic Process

Specific structural factors facilitated our autoethnographic processes. The first is the self-initiated, self-governing nature of the project, forcing the project group to generate and negotiate its own objectives, procedures, and self-understanding (Table 1, row 6). Out of these discussions, the collective project narrative and shared mental model were generated. Having to present it regularly to project participants and stakeholders led to it being continuously updated and spread internally and externally, becoming the project’s ongoing oral history (Ong, 1982; Silva, 2021). Without this structural continuous demand to present and account for the project, the campfire narrative would not exist in its living form. The oral modality is a result of oral communication being a central practiced skill in the surgical profession, and the opportunity or demand for regular oral project accounts throughout a year. The memory process behind the oral narrative is supported by the test-days always taking place in the same facilities. Research on location and memory, especially workplace contexts, shows locational continuity aids embodied and embedded “circuits of memory” (Pleasant & Strangleman, 2019). This well-rehearsed, dynamic narrative can then be exported to other locations, such as conference and stakeholder presentations, or in written form for funding and ethics vetting bodies.

### Our Adaptation of Autoethnography

Our autoethnoral practice deviates from the predominant autoethnographic form, especially if defined as evocative, text-based, individual autoethnography rooted in the academic world. Based on the preceding discussions, Table 1 summarizes the most significant deviations, advantages, and recommendations of our adaptation.

As discussed in the Analytical Versus Evocative Autoethnography section, there is a reticence about portraying and acting on others found in the “evocative” version of autoethnography (Table 1, row 2). Though the “problem of representation” is seen by adherents of evocative autoethnography as absolutely essential (Denzin, 1997; Ellis et al., 2011), analytic autoethnography rejects this imperative, seeing analytic autoethnography as a basis for direct practical interventions and contextual change (Acosta et al., 2015; Lapadat, 2017). Orality doesn’t solve but lessens concerns about representations. Oral representations, albeit having effects, remain fluid and readily subject to revision and dialogic interventions, not the least from the represented parties.

As the objective of our project is not just understanding a contextual situation but also producing an improved procedure for resident selection, we make categorical assessments and act from a position of power and privilege, impacting the fates of those who aspire to surgical residency positions in Sweden. Furthermore, as evidenced above, our process is explicitly oriented toward uncovering our biases, assumptions, partial understandings, and increasing our awareness of the positions of power and privilege we individually and collectively possess and exercise (Table 1, row 4). Here, we clearly depart from evocative autoethnography’s moral position of compelling persuasion for voluntary action in favor of using analytical autoethnography as a means of producing actionable knowledge beyond the individual level—at the organizational and structural levels. Furthermore, autoethnography is used to increase our reflexive understanding (Acosta et al., 2015; Silva, 2021) in the exercise of hierarchical power to improve patient safety, medical outcomes, and the work environment.

### Making Autoethnography More Amenable to Practitioner Contexts: From Autoethno-graphy to Autoethn-*orality*

We conclude this section by explicitly addressing how deeper engagement with practitioners and practice will modify autoethnography. Like others (Acosta et al., 2015; Chang et al., 2013; Lapadat, 2017), we find CAAE highly beneficial in facilitating our collaboration. The collaborative dimension opens up for both more rigor and interdisciplinary perspectives (Lapadat, 2017). Multi- and interdisciplinarity and academic–practitioner interaction calls for reconciling different and even apparently conflicting perspectives. As recounted in the narratives above, this is precisely one of the strengths of autoethnography, bringing what at one level are intractable conflicts down to where they can be broached and brokered at the level of personal experience.

If autoethnography’s objective is facilitating reflexion to produce actionable knowledge in hierarchical, organizational settings (Acosta et al., 2015; Silva, 2021), analytic autoethnography with its realism and engagement beyond the self provides a viable foundation. Here, one has to sacrifice evocative autoethnography’s voluntarism and evading resolution to the “problem of representation” (Denzin, 1997; Ellis et al., 2011). Furthermore, for autoethnography to gain greater traction in practitioner contexts, it needs to build on the reflective practices, skillsets, and timeframes indigenous to the practitioner world rather than academia. We contend that a main challenge for autoethnography as it moves into the world of practice is to develop methods and practices that recognize and function in the modality of orality. This may ultimately lead to the establishment of a complementary methodology of autoethn*orality*, such as it is described in this article.

### Limitations, Credibility, and Transferability

This article confronts two skepticisms inherent in the mainstream, positivist medical science paradigm. The first has to do with self-generated observations of oneself and processes one engages in. This runs counter to the hegemonic observer–object relation at the core of the positivistic scientific method, including traditional ethnography. However, as Anderson (2006) argues, autoethnography can proceed on realist foundations. Furthermore, as discussed above with reference to Lapadat (2017), the collaborative autoethnographic approach adopted here corrects the most tenuous aspects of individual autoethnography, generating collective systematic qualitative knowledge about proximate contexts.

The second skepticism is likely more pervasive. This has to do with the scientific status of oral sources and processes. Science is based on documentation—recorded observations and written records. To be scientific (and publishable), we have had to “textualize,” primarily in account form, essentially oral content and oral processes. As discussed above, there are means to work in a hybrid manner, blending orality with different means of documentation. Furthermore, this article immanently straddles two worlds—academic research and healthcare practice. The scientific criteria of validity and reliability are brokered alongside the practitioner’s criteria of relevance and practical value. The primary purpose of this article is to report on autoethnoral techniques found through the collective experience of the research group to assist in promoting positive group dynamics, overcoming paradigmatic theory of science discrepancies, and generating deep and novel insights into our proximate context. Along with Patterson et al.’s (2018) statement about the need to move beyond mono-methodological and mono-paradigmatic approaches, we believe that the development of adapted autoethnographic methods is meaningful to others, especially those involved in similar projects investigating complex phenomena requiring multi-disciplinary collaboration with risk for clashing scientific paradigms.

## Concluding Reflections

Our empirical case in the healthcare sector illustrates the benefits and challenges autoethnorality offers for increasing reflexivity and resolving tensions in multi-disciplinary, academic–practitioner collaboration. Particularly, CAAE has been shown to produce both reflexive self-awareness and contextual insights. Autoethnography, with its origins in academic research strongly wedded to text-based reflexion and production (Ong, 1982; Lapadat, 2017) rather than the timeframes, concerns, skillsets, and practices of practitioners, will need to change to become more resonant and practicable in practitioner environments. Modalities of orality will need to be recognized and further developed as part of the autoethnography toolkit to facilitate uptake in practitioner communities.

Autoethnorality/graphy can also be catalyzed by common reading and discussion of boundary-crossing literature to facilitate discussions on paradigms and pre-formed perceptions based on different types of scientific education. This opens for moving from multi-disciplinarity to trans-disciplinarity through reflexivity.

This article and our empirical case show there are many ways of practicing autoethnography. We find the collaborative analytic variant, especially locally adapted and recognizing orality, is most promising and amenable in facilitating deeper scholar–practitioner collaboration and reflexive practice in practitioner settings.

## Note

1. Keyhole abdominal surgery.
